# Host-Pathogen Interaction as a Novel Target for Host-Directed Therapies in Tuberculosis

**DOI:** 10.3389/fimmu.2020.01553

**Published:** 2020-07-21

**Authors:** Rodrigo Abreu, Pramod Giri, Fred Quinn

**Affiliations:** Department of Infectious Diseases, University of Georgia, Athens, GA, United States

**Keywords:** lipid metabolism, iron metabolism, macrophage, tuberculosis, *Mycobacterium*

## Abstract

Tuberculosis (TB) has been a transmittable human disease for many thousands of years, and *M. tuberculosis* is again the number one cause of death worldwide due to a single infectious agent. The intense 6- to 10-month process of multi-drug treatment, combined with the adverse side effects that can run the spectrum from gastrointestinal disturbances to liver toxicity or peripheral neuropathy are major obstacles to patient compliance and therapy completion. The consequent increase in multidrug resistant TB (MDR-TB) and extensively drug resistant TB (XDR-TB) cases requires that we increase our arsenal of effective drugs, particularly novel therapeutic approaches. Over the millennia, host and pathogen have evolved mechanisms and relationships that greatly influence the outcome of infection. Understanding these evolutionary interactions and their impact on bacterial clearance or host pathology will lead the way toward rational development of new therapeutics that favor enhancing a host protective response. These host-directed therapies have recently demonstrated promising results against *M. tuberculosis*, adding to the effectiveness of currently available anti-mycobacterial drugs that directly kill the organism or slow mycobacterial replication. Here we review the host-pathogen interactions during *M. tuberculosis* infection, describe how *M. tuberculosis* bacilli modulate and evade the host immune system, and discuss the currently available host-directed therapies that target these bacterial factors. Rather than provide an exhaustive description of *M. tuberculosis* virulence factors, which falls outside the scope of this review, we will instead focus on the host-pathogen interactions that lead to increased bacterial growth or host immune evasion, and that can be modulated by existing host-directed therapies.

## Tuberculosis Epidemiology

Despite extensive efforts to control *Mycobacterium tuberculosis* infections through robust screening and therapeutics programs, the World Health Organization (WHO) reported over 10 million new cases in 2018, with over 1.5 million fatalities, ranking as the leading infectious killer in the world, surpassing HIV in 2017 (1). Worldwide incidence of tuberculosis (TB) has been slowly falling over the last 15 years at an average rate of 1.5% per year and prevalence is estimated to have fallen 42% between 1990 and 2015. Nonetheless, TB incidence remains high in Asia, India and Africa (2). In addition to the high number of active TB cases, approximately one third of the world population is estimated to have latent TB infection with 10% having a lifetime risk of developing active infection (3). With the lack of more sensitive and specific diagnostic tools, latent TB infection is typically identified by a positive immune response to *M. tuberculosis* antigens (tuberculin skin test or interferon-gamma release assay) in the absence of clinical manifestations. HIV co-infection or immunosuppressive treatment (anti-TNF-α or transplant patients) significantly increases the risk of reactivation to 10% chance every year (2). Out of the 9.6 million TB cases in 2014, more than one million were HIV-positive with about 35% resulting in death. There was a higher incidence rate in Africa where over 30% of all TB cases are in HIV co-infected patients (4).

**Figure 1:**
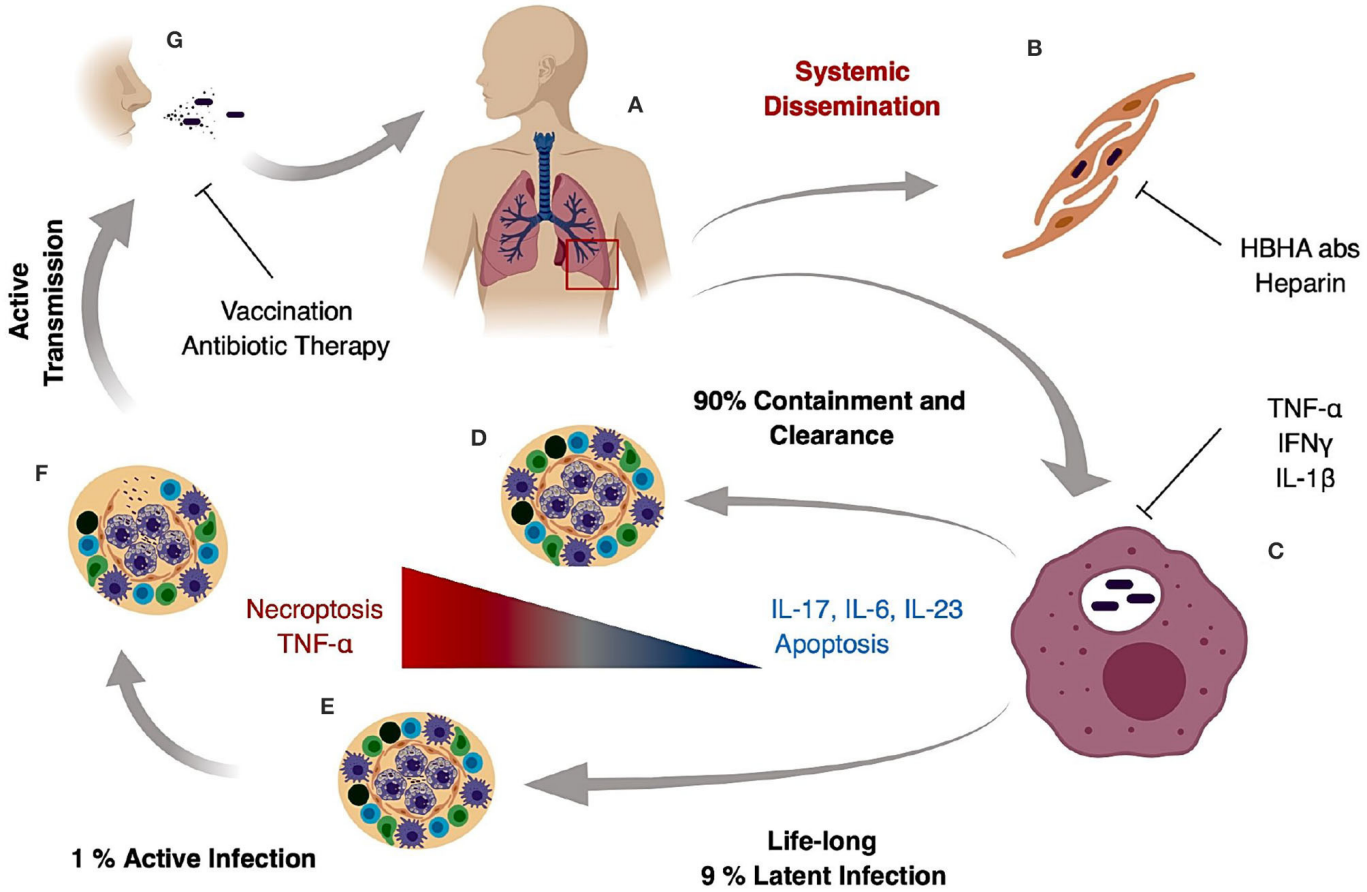
Tuberculosis infection and transmission hallmarks. Inhaled *M. tuberculosis* bacilli travel to the alveoli where they are phagocytized by alveolar macrophages **(A)**. It is hypothesized that internalization and successful replication within Type II pneumocytes results in systemic dissemination and extrapulmonary TB, which can be decreased by HBHA neutralizing antibodies or heparin treatment **(B)**. In the lung, *M. tuberculosis* bacilli replicate in alveolar macrophages during early stages of infection **(C)** and in 90% of the cases the host mounts an appropriate immune response preventing pathogen entry or controlling pathogen growth and replication resulting in bacterial clearance **(D)**. In 9% of the cases, the host develops a life-long latent stage with bacterial containment most prominently maintained inside caseous granulomas **(E)**. In latent cases, a 10% lifetime risk of reactivation due to an improper immune response or immunosuppression can occur. The result is loss of granuloma integrity, *M. tuberculosis* growth, dissemination, and ultimately infection of the upper lobes **(F)**. Uncontrolled bacterial replication and granuloma caseation **(F)** augments lung pathology and initiates active aerosol transmission to the next host **(G)**.

*M. tuberculosis* generates systemic infection but is primarily identified in adults as a lung pathogen that interacts to a significant extent with alveolar macrophages and if not cleared, leads to extensive lung inflammation, dissemination and pathology. If active disease develops, symptoms are characterized by persistent cough that can last for several weeks, late day fevers (night sweats), constant fatigue, loss of appetite, and severe weight loss (1, 5, 6). Infection with *M. tuberculosis* primarily is caused by inhalation of bacilli, transmitted by an actively infected individual. The inhaled bacilli can progress in different stages depending on the host immune system (Figures 1A,B). In 90% of primary infected individuals the host is capable of controlling and resolving the infection (Figure 1D). In latent infection which occurs in ~7–10% of infection cases, mycobacterial replication is minimal and primarily contained in small granulomatous structures until re-activation. Clearance may take up to 3 years, but in some cases it never occurs and the pathogen goes into a life-lasting latent stage that can reactivate in case of immunosuppression (7) (Figure 1E). In primary active TB, *M. tuberculosis* bacilli migrate to the alveoli where they encounter alveolar macrophages and dendritic cells that actively phagocytize the bacteria and ultimately the bacilli and/or infected phagocytes disseminate to regional lymph nodes (Figure 1C). This first stage can take 3–8 weeks or longer and has no clear manifestation or transmission stage. In a second phase that can last up to 3 months after primary infection, hematogenous dissemination of the bacteria leads to *M. tuberculosis* spread into the upper and lower lobes of the lung and can cause systemic dissemination including meningitis TB which in many cases is fatal (7) (Figure 1B).

Traditional research in *M. tuberculosis* virulence focused on the comparison of virulent laboratory or clinical strains against the attenuated *M. bovis* BCG vaccine strain (8). Genetic analysis of *M. tuberculosis* lab strain H37Rv against BCG revealed 14 regions of differentiation (RD1–RD14) of which three (RD1, RD2, and RD14) are still present in clinical strains of *M. bovis* (9–12). Within RD3–RD13, multiple genes have been associated with *M. tuberculosis* virulence both *in vivo* and *in vitro*, and have been extensively reviewed elsewhere (12). In this review, we will focus on the host-pathogen interactions that lead to increased bacterial growth in the host that can be modulated by existing host-directed therapies (HDT).

## Drug Treatment and Drug Resistance

Drug treatment for TB requires complex drug regimens for long periods of time leading to severe side effects. WHO guidelines recommend the treatment of newly diagnosed TB cases with a six-month regimen of isoniazid, rifampicin, pyrazinamide, and ethambutol during the intensive phase (first 2 months) followed by isoniazid and rifampicin for continuation phase (next 4 months) (13). In cases of TB relapse with a medium- to low-risk of multidrug-resistance the addition of streptomycin to the abovementioned drug regimen during the intensive phase is recommended, followed by a 1-month regimen of isoniazid, rifampicin, pyrazinamide and ethambutol, and a 5-month regimen of isoniazid, rifampicin, and ethambutol (4, 6).

Between 2005 and 2013, only 86% of treatments for newly diagnosed TB cases were successfully completed. This lack of therapy compliance leads to an increase in MDR-TB and XDR-TB cases. In 2014, 3.3% of all new TB cases and 20% of previously treated cases were MDR-TB, accounting for a total of almost 500,000 patients worldwide (2). This increases urgency for the development of new therapeutic strategies through the discovery of new anti-mycobacterial drugs and the identification of HDT that provide a hostile environment for the growth of the organism and promote a protective immune response (4). In fact, novel therapeutic approaches largely centered on host pharmacological targets have been recently reviewed and the focus of grand clinical interest (14–16), but fail to cross-reference with the target bacterial factors. Here we review the known host-pathogen interactions during *M. tuberculosis* infection, how the bacteria modulate and evade the host immune system, and the currently available HDT that target each mechanism (Table 1).

**Table 1:** Currently available host-directed therapies for tuberculosis.

**Compound**	**Host-pathogen interaction**	**Mechanism of action**	**Phase**	**References**
*M. bovis* BCG HBHA vaccines	Systemic dissemination Extrapulmonary infection	Inhibits HBHA-mediated adherence and internalization of Type II pneumocytes	Approved for human use Clinical optimization	(17) (18)
Glucocorticoids (dexamethasone)	Systemic dissemination Extrapulmonary TB meningitis	Decreased inflammation and other unknown effects	Approved for human use	(19–21)
Heparin	Systemic dissemination Extrapulmonary infection	Inhibits HBHA-mediated adherence and internalization of Type II pneumocytes	Approved anticoagulant therapy	(22)
	Modulation of macrophage iron status	Inhibits Hepcidin expression and intracellular iron sequestration	Preclinical research and development	(23)
Vitamin D3	Macrophage anti-microbial functions	Induces phagolysosome fusion and autophagy in macrophages	Clinical optimization	(24)
MicroRNA therapy miR-33, 144-3p, 155, 146a, 20a-5p	Macrophage anti-microbial functions Granuloma formation and pathology	Regulation of apoptosis, TLR signaling, RNS, VD3 induced genes and TNFα	Preclinical	(25–34)
Metforin	Macrophage anti-microbial functions	Induces ROS and RNS production, reduces glycolysis and Mtb-induced foamy cell differentiation	Ready for clinical trials	(35, 36)
Defensins	Anti-microbial activity, activation of adaptive immune system	Direct bacterial lysis, cellular chemotaxis of macrophages, DC and T-cell	Unsuccessful in clinical trials, preclinical	(37–40)
Imatininb	Modulates *M. tuberculosis* uptake Macrophage anti-microbial functions	Induces phagolysosome fusion and autophagy in macrophages	Preclinical research and development	(41, 42)
PRR agonist	Activation of adaptive immune system, macrophage anti-microbial functions, modulation of macrophage iron status	Induces cytokine secretion, phagosome maturation, autophagy, ROS and RNS production	Clinical optimization	(43, 44)
Statins (rosuvastatins)	Modulates macrophage lipid metabolism	Inhibits cholesterol synthesis, lipid accumulation in macrophages and foamy cell differentiation	Ready for clinical trials	(45–47)
Hepcidin inhibition	Modulates macrophage iron status	Inhibits hepcidin-mediated ferroportin degradation and intracellular iron sequestration in macrophages, M1 polarization	Preclinical research and development	(48–50)
Anti-TNFα	Decreases pathology and granuloma caseation	Inhibits necrosis of infected cells in the granuloma center	Failed in trials	(51)
Cytokine therapy (IFNγ, IL-17)	Activates adaptive immune system	Induces T_H_1 and T_H_17 adaptive immune response	Ready for clinical trials	(52, 53)
NSAIDs (ibuprofen)	Decreases pathology and granuloma caseation	Induces expression of anti-inflammatory eicosanoids and apoptosis of infected cells in the granuloma center	Ready for clinical trials	(54)
Zileuton (asthma drugs)	Decreases pathology and granuloma caseation	Induces apoptosis of infected cells in granuloma center	Ready for clinical trials	(51, 55)

## Systemic Dissemination

*M. tuberculosis* is primarily known as a pulmonary pathogen; however, it is commonly found to cause disseminated disease with lesions present in many organs and tissues including the spleen, lymph nodes and brain. It can also manifest as a more generalized disseminated form known as miliary TB. The mechanisms involved in dissemination from the lung are not well understood, but *M. tuberculosis* entry, intracellular replication and necrosis of alveolar epithelial cells is thought to be one mechanism involved in extra-pulmonary dissemination. Attachment to Type II alveolar epithelial cells (pneumocytes) is likely mediated by several bacterial adhesins. One adhesin that has been well studied is the heparin-binding hemagglutinin (HBHA) (18, 22, 56). Inhibiting invasion of Type II pneumocytes with heparin and heparin sulfate, or blocking HBHA function with neutralizing antibodies efficiently prevents *M. tuberculosis* dissemination (17). Another rare form of extra-pulmonary TB, most prevalent in young children, results in meningitis, the most lethal form of *M. tuberculosis* infection. *M. bovis* BCG vaccination remains the main prophylactic approach against TB meningitis with almost 80% protection against this form of disease in young children (57). In fact, meningitis protection in children is one of the main reasons for continuing widespread BCG vaccination programs particularly in endemic areas even in the absence of efficient protection against pulmonary TB. The exact protection mechanisms elicited by *M. bovis* BCG remain elusive, but elicitation of strong humoral responses against HBHA and other *M. tuberculosis* surface proteins along with long lasting cellular responses to overlapping internal antigens have been shown to prevent extra-pulmonary dissemination, and consequently meningitis. Nonetheless, in case of dissemination adjunctive glucocorticoid therapy with standard of care anti-mycobacterial drug regiments increases TB meningitis survival rates (19–21).

## Macrophage Roles During Infection

Inhibiting entry into target host cells by intracellular pathogens is a frequent therapeutic approach that limits disease pathogenesis. During *M. tuberculosis* infection this strategy is particularly difficult since a major cell target, alveolar macrophages, are also a crucial player in the host immune response. Upon attachment, alveolar macrophages actively phagocytize *M. tuberculosis* bacilli through multiple mechanisms, and the internalization pathway greatly influences microbiocidal efficiency (58–60).

During early primary *M. tuberculosis* infection, direct mycobacterial phagocytosis is mostly mediated by C-type lectin receptors (CLRs) (61, 62). The macrophage mannose receptor (MMR) recognizes *M. tuberculosis* lipoarabinomannan (63, 64) and is predicted to signal through a putative cytoplasmic tyrosine domain, which phosphorylates and activates CDC42, RHOB, PAK, or ROCK1, involved in actin reorganization, membrane invagination and phagosome formation (62, 65–68). Another CLR involved in *M. tuberculosis* recognition by macrophages is macrophage-inducible C-type lectin which recognizes trehalose-6,6-dimycolate, an abundant mycobacterial cell wall glycolipid (12, 59, 69). In later stages of infection or in secondary infections, antibody and complement opsonized bacteria are phagocytized through Fc and complement receptors, signaling through a similar mechanism that promotes efficient bacterial killing and controls replication (60, 62). Despite the importance of these receptors in *M. tuberculosis* cell attachment and phagocytosis, the impact of each mechanism in the outcome of infections is not yet clear. Recently a tyrosine kinase inhibitor used in cancer therapy has been shown to modulate *M. tuberculosis* uptake and promote bacterial killing *in vitro* and *in vivo* (41, 42). Moreover, this drug was particularly effective in combination with anti-mycobacterial drugs, but the exact mechanism remains elusive. It is possible that a decrease in bacterial internalization by macrophages increases antibiotic access to the bacilli, or that inhibition of one specific internalization pathway leads to an alternative uptake mechanism that activates microbiocidal macrophage functions. Currently, Imatinib is the only tyrosine kinase inhibitor tested as a modulator of *M. tuberculosis* invasion, but other similar drugs presently in trials for cancer therapy (42) might have similar impacts or help clarify the exact mechanism behind bacterial control *in vivo*.

## Granuloma Formation and Pathology

A hallmark of *M. tuberculosis* infection and pathology is granuloma formation and maintenance. The granuloma is a compact organized immunological structure built of macrophages, monocytes, dendritic cells, neutrophils, epithelioid cells, foamy macrophages, and multi-nucleated giant cells, enclosed by T and B lymphocytes (70). Disease progression results from complex remodeling of the granuloma structure with increased hypoxic necrotic centers rich in lipids and foamy macrophages that fail to control bacterial replication ultimately leading to granuloma caseation (70). The mycobacterial factors leading to granuloma restructuring and rupture are not yet well-described, but the ESX-1 secretion system including ESAT6, and TDM are known to play important roles in the initial steps of granuloma formation (12, 71). Alternatively, TNF-α, IL-6 and complement (C5) are important for cellular recruitment and maintenance of the granuloma structure. In the granuloma center, predominant apoptotic cell death of infected macrophages controls bacterial replication by efferocytosis (72). In contrast, necrosis results in bacterial leakage into the growth permissive extracellular environment, and a characteristic cording phenotype hampers phagocytosis by newly recruited macrophages (70, 71, 73). Efficient *M. tuberculosis* infection strongly modulates macrophage cell death. In human primary macrophages, *M. tuberculosis* bacteria significantly modulate expression of microRNAs (miR−145 and miR-20a-5p) regulators of apoptosis, remodeling cell death toward a necroptotic pathway (25, 26, 29).

Efficient bacterial control in the granuloma requires a balanced pro- and anti-inflammatory environment (74). Anti-TNF-α therapy in patients with autoimmune disorders has been shown to increase the risk of TB reactivation (75); however, excessive TNF-α leads to increased macrophage necrosis that results in granuloma caseation (72, 76, 77). Central in the regulation of TNF-α expression during *M. tuberculosis* infection are pro-inflammatory eicosanoids such as leukotrienes and prostaglandins (51, 55). Excessive leukotrienes promote TNF-α and Type I IFNs that result in increased necrotic cell death, granuloma caseation and cavity formation (55). Alternatively, IL-1 signaling promotes apoptosis and induces prostaglandin expression which counter-regulates the function of Type I IFN (51, 55). Non-steroid anti-inflammatory drugs, such as ibuprofen, induce expression of anti-inflammatory eicosanoids that significantly ameliorates pathology during *M. tuberculosis* infection *in vivo* with reduced bacterial load (54). Similarly, leukotriene inhibitors such as Zileuton used for asthma therapy, also reduce bacterial load in *M. tuberculosis* susceptible animal models (51, 55). Finally, new therapeutic approaches targeting pathologically imbalanced microRNAs are rapidly arising for cancer and inflammatory diseases (78–80). Similar approaches targeting miR-145 and miR-20a-5p might counteract *M. tuberculosis* anti-apoptotic effects favoring granuloma integrity and bacterial clearance.

## Modulation of the Host Adaptive Immune Response

Despite extensive research, it is not yet clear what might be the ideal adaptive immune response leading to efficient control of bacterial replication and clearance with minimal tissue damage (59, 81). *Mycobacterium tuberculosis* bacteria infect professional antigen-presenting cells with a significant impact on antigen presentation and activation of the adaptive immune response. Dendritic cells infected with *M. tuberculosis* bacilli have decreased MHC surface expression and impaired antigen processing and presentation to ${\text{CD}}_{4}^{+}$ T cells (82, 83). The priming of T helper cells is delayed by *M. tuberculosis*, and modulation of cytokine secretion by macrophages promotes differentiation of Tregs and secretion of decoy antigens that modulate the humoral response (84, 85). ${\text{CD}}_{4}^{+}$ T cell activation and differentiation into T_H_1, with IL-2 and IL-12, and into T_H_17 subsets, with IL-6, IL-1β, and IL-23, is essential for *M. tuberculosis* containment (84). Thus, the effector cytokines produced by these two T helper cell subsets have long been hypothesized as an effective immunomodulatory host-targeted therapy for TB. Despite the long recognized importance of IFNγ producing T_H_1 ${\text{CD}}_{4}^{+}$ cells for an effective adaptive immune response (86, 87), direct IFNγ therapy produced controversial results in TB patients (52). Initial studies with non-tuberculosis mycobacteria-infected patients (atypical pulmonary mycobacteriosis), showed that IFNγ treatment in combination with standard anti-mycobacterial chemotherapy had no impact on sputum cultures, but a pronounced effect in treatment completion rates and decreased lung lesion severity were observed (88). A similar IFNγ treatment study with pulmonary TB patients showed no significant changes in disease morphology as observed by chest radiology results, however the IFNγ treatment did attenuate general disease symptoms such as fever and increased rates of sputum smear conversion (52). Furthermore, other direct cytokine therapies with IL-2 or IFNα also failed to produce conclusive beneficial results during *M. tuberculosis* infection (53) indicating that single direct cytokine therapy might not be sufficient alone as a HDT approach for anti-TB treatment. Recent studies highlighting the importance of multifunctional T_H_1 cells capable of producing multiple cytokines (IL-2, TNF-α, and IFNγ) might explain this discrepancy between the importance of some cytokines for an effective host immune response and the inefficacy of these same cytokines in clinical trials (89–91). Instead of direct adaptive immune activation, HDT can also target chemotaxis of innate immune mediators. Defensins are strong chemoattractants of macrophages, dendritic cells and T-cells and promote T_H_1 responses (37–40). Furthermore, defensin therapy would also directly target extracellular bacteria if administered intranasally (92). Finally, other immunomodulatory therapeutic approaches, focus on Treg downregulation. Infection with *M. tuberculosis* bacilli promote a tolerogenic immune response and the differentiation of Tregs to facilitate bacterial replication (59). GR1-specific antibodies and denileukin/diftitox efficiently deplete Treg proliferation and other myeloid-derived suppressor cells and significantly enhance anti-mycobacterial drugs effects (93, 94). This is a very active area of research particularly in anti-cancer therapy, but must be approached carefully because breaking host tolerance is frequently associated with severe autoimmune diseases.

## Macrophage Activation Signaling

Innate immune cells like macrophages or dendritic cells recognize a myriad of pathogen or danger associated molecular patterns (PAMPS or DAMPS) (62). Efficient microbiocidal functions in macrophages require activation of these stimulatory pathogen recognition receptors (PRR) such as Toll-like (TLR) or Nod-like (NLR) receptors (62). *Mycobacterium tuberculosis* bacilli evade and modulate PRR signaling to promote recruitment of permissive macrophages and manipulate the host adaptive immune response (58, 59, 95, 96).

Toll-like receptors are abundantly expressed in human macrophages and crucial for early pathogen recognition during infection (97). The relevance of TLR signaling for *M. tuberculosis* containment is still being assessed (98–100), but it is widely recognized that virulent *M. tuberculosis* strains modulate and evade TLR signaling (58). Non-pathogenic mycobacterial cell wall glycolipids such as lipoarabinomannan strongly activate TLR2 signaling inducing a strong pro-inflammatory response (101, 102). Contrastingly, similar molecules from *M. tuberculosis* such as mannose-capped lipoarabinomannan do not activate TLR2 signaling or induce pro-inflammatory cytokines (58, 64). Recent studies focusing on post-transcriptional regulation by microRNA start to shed light on how *M. tuberculosis* can modulate TLR signaling (27), but the promiscuous activity of microRNAs and the myriad of targets altered during *M. tuberculosis* infection obstruct the design of a definitive model.

*M. tuberculosis* bacilli interfere with phagosome maturation, compromise phagosome membrane integrity (103, 104) and some reports describe the bacilli escaping the phagosome and residing in the cytoplasm (105–107). Nod-like receptors are crucial to recognizing cytosolic PAMPS during bacterial infection and play an important role in inducing Type I IFN and inflammasome activation (108). NOD2 recognizes bacterial muramyl dipeptide fragments of the cell wall peptidoglycan in the cytosol and induces autophagy and pro-inflammatory cytokine production (43). However, *M. tuberculosis* muramyl dipeptides are N-glycolyl modified and modulate NOD2 signaling to an alternative pathway leading to production of Type I IFNs which are not protective during *M. tuberculosis* infection (109). Furthermore, Type I IFNs antagonize IL-1β and IFNγ host-protective signaling (110).

The use of PRR ligand adjuvants is a particularly active area in vaccine development (111–113), but the use of specific TLR or NLR agonists might also be useful as a HDT. Activation of TLR2 with its specific ligand Pam2Cys rescues T_H_1 cell exhaustion and significantly ameliorates disease in chronically *M. tuberculosis*-infected mice (44). Similarly, NOD2 and TLR4 activation significantly enhances the effect of standard anti-mycobacterial drugs isoniazid and rifampicin in *M. tuberculosis*-infected dendritic cells (43). Alternatively, modulating the expression and activity of miR-146a and miR-155, two microRNAs extensively described as regulators of innate immune cell activation downstream of TLR activation might favor macrophage antimicrobial functions (27). These studies, although preliminary, show the potential of direct PRR activation as an immunomodulatory HDT for TB. Nonetheless, such therapeutic approaches must proceed with care since dysregulated PRR signaling is frequently associated with loss of immune tolerance and as described earlier, development of autoimmune diseases.

## Inhibition of Macrophage Microbiocidal Functions

**Figure 2:**
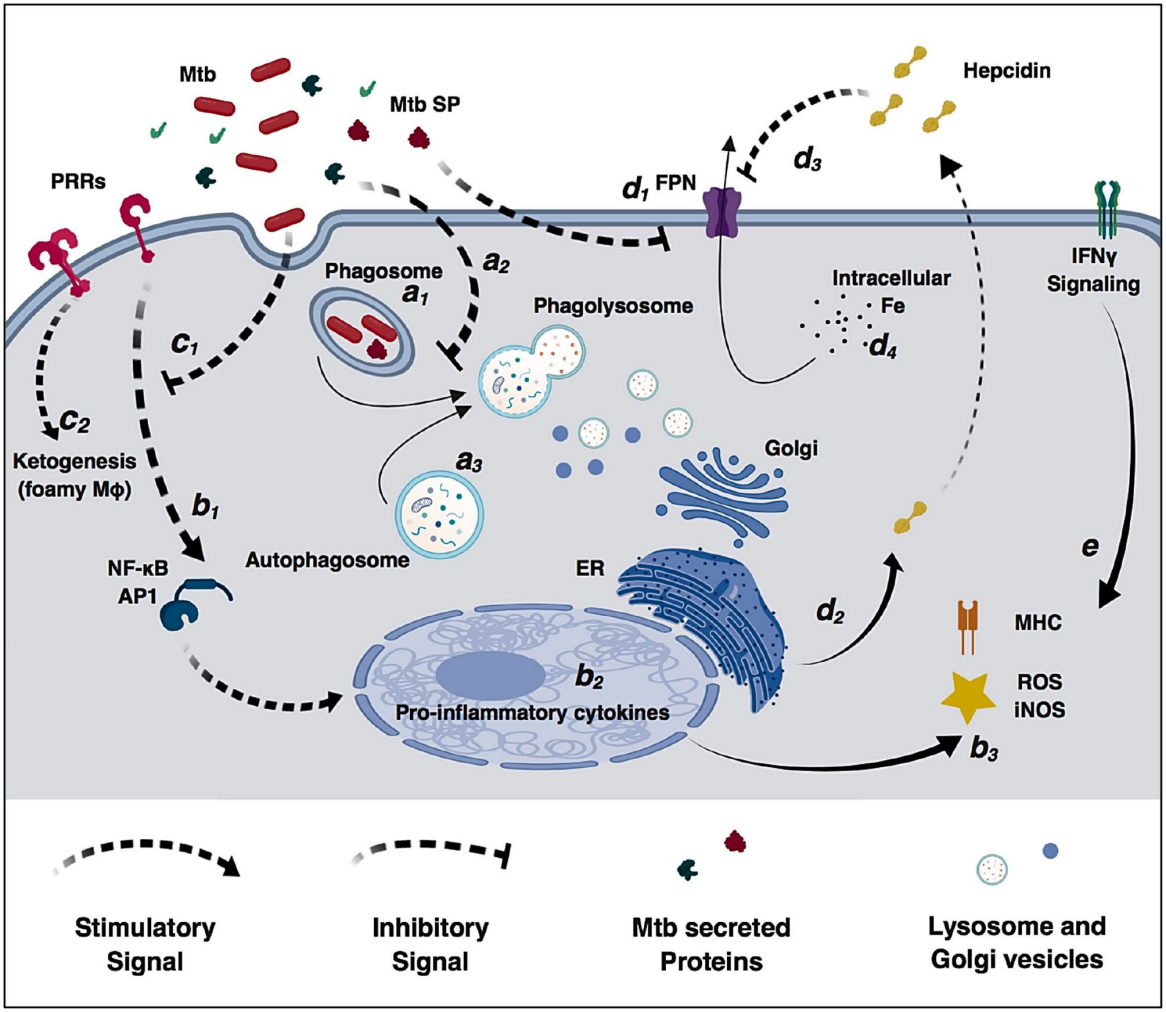
Modulation of macrophage immune functions by *M. tuberculosis* (*Mtb*). Bacilli are phagocytized by macrophages through different surface receptors **(a**_**1**_**)** which greatly influence phagosome maturation and lysosome fusion **(a**_**2**_**)**. *M. tuberculosis* secreted proteins further inhibit phagosome fusion, but autophagy induction redirects immature phagosomes to the autophagosome **(a**_**3**_**)** increasing bacterial killing. Macrophages detect pathogen invasion through activation of pathogen-recognition receptors (PRRs) **(b**_**1**_**)** leading to expression of pro-inflammatory cytokines **(b**_**2**_**)**, increased reactive oxidizing species and activation of the adaptive immune system **(b**_**3**_**)**. However, *M. tuberculosis* cell-wall glycolipids modulate PRRs signaling **(c**_**1**_**)**, increase lipid accumulation, promote the differentiation in permissive foamy cells **(c**_**2**_**)** and inhibit cytokine secretion. Infection in macrophages directly decreases ferroportin transcriptional expression **(d**_**1**_**)**, and *M. tuberculosis*-induced endoplasmic reticulum stress induces hepcidin expression and secretion **(d**_**2**_**)**. Secreted hepcidin binds to ferroportin leading to its internalization and degradation **(d**_**3**_**)**. Decreased surface levels of the iron exported by ferroportin result in increased intracellular iron sequestration in macrophages **(d**_**4**_**)** that can be redirected to the immature phagosome and used by *M. tuberculosis* for replication. IFNγ signaling increases macrophages antimicrobial functions and counteracts *M. tuberculosis* immunomodulatory mechanisms **(e)**.

Alveolar macrophages are an important cell target for *M. tuberculosis* infection. In an ideal immune response, macrophages efficiently phagocytize and control bacterial replication. In this scenario, phagosomes containing live mycobacteria fuse with lysosomes from the Golgi apparatus that lead to an acidified environment, increased reactive oxygen species (ROS) and reactive nitrogen species (RNS) species, and high protease activity (Figure 2). These processes culminate in bacterial killing and clearance (61, 101). However, *M. tuberculosis* can subsist and replicate inside macrophages by interfering with phagosome maturation and blocking the macrophage microbiocidal mechanisms (65). Generally, *M. tuberculosis* resorts to three different mechanisms to prevent phagosome killing: phagosome maturation arrest, phagosome evasion, and oxidative and nitrosative stress neutralization.

Several proteins expressed by *M. tuberculosis* are capable of inhibiting or blocking phagosome maturation and phagolysosome fusion; e.g., nucleoside diphosphate kinase, a 14 kDa protein isolated from the culture medium, interacts and inactivates Rab7 and Rab5 which are crucial for phagosome-lysosome fusion (12, 60, 114, 115). Similarly, phosphotyrosine protein A (PtpA), a low molecular weight phosphatase, can bind and block the host vacuolar H^+^-ATPases and dephosphorylate a host vacuolar protein preventing phagosome acidification and maturation (116–118). Aside from these, many other mycobacterial factors have been associated with phagosome maturation or arrest and extensively reviewed elsewhere (12, 60). Until now, IFNγ activation and autophagy induction seem to be the most promising pathways to promote phagosome maturation and phagolysosome fusion (119–121). *In vitro* macrophage activation with recombinant IFNγ upregulates FcR and CR3 surface expression (122–124), favoring phagocytosis of opsonized bacilli. As mentioned above, this phagocytic pathway promotes phagosome acidification and phagolysosome fusion. The protective effect of vitamin D3 during TB has long been recognized but the mechanisms involved remained elusive (125). Now, we realize that vitamin D3 induces cathelicidin expression in macrophages, an antimicrobial peptide important in phagosome maturation and phagolysosome fusion (24). Furthermore, has-mir-21 has been shown to play a central role in vitamin D3-dependent cathelicidin expression following bacterial infection (126), suggesting that modulation of this specific microRNA might serve as a valuable HDT target. Likewise, imatinib promotes phagosome maturation, lysosome fusion and autophagy (41), a naturally occurring cellular process for recycling and degradation of cytosolic content through vesicular engulfment and lysosome fusion (127). During *M. tuberculosis* infection, phagosomes containing live bacilli are redirected to the autophagy pathway reactivating lysosome fusion and bacterial killing (119), and here too, microRNAs (miR33, miR-155, and miR144-3p) have been shown to play a crucial role in autophagy regulation (28, 32, 33). Another possible target is the NAD^+^-dependent histone deacetylase sirtuin 1 (SIRT-1), which was recently shown to be downregulated during *M. tuberculosis* infection but important for controlling bacterial replication (128). Resveratrol is a phytoalexin present in grapes and berries, frequently commercialized as food supplement and a natural SIRT-1 activator. Resveratrol and a synthetic SIRT-1 activator induce phagolysosome fusion and autophagy, restricting *M. tuberculosis* growth *in vitro* and *in vivo*. Anti-mycobacterial drugs shown to induce autophagy with minimal cell toxicity are a very active HDT research area targeting viral and bacterial infections (119, 129).

For decades intracellular *M. tuberculosis* bacilli were believed to merely inhibit phagosome maturation, growing and replicating inside this vesicular structure and not escaping into the cytoplasm (63, 84, 130, 131). However, recently *M. tuberculosis* bacilli have been associated with complete phagosome evasion through permeabilization of the phagosome membrane similar to *Shigella* or *Listeria* (105). ESAT6/CF10 proteins, secreted by ESX-1 T7SS, have cell membrane lysis properties (132) and likely are involved in bacterial escape from the phagosome to the cytoplasm in dendritic cells (12, 133, 134). Currently, there are no prospective therapies to target cytosolic bacilli and prevent phagosome evasion, but modulation of the host ubiquitination machinery, inducing autophagy and activating cytosolic PRRs have been shown important for containment of other cytosolic pathogens (135, 136).

Oxidative and nitrosative stress play a crucial role in bacterial clearance in macrophages. In these cells, NOX2 NADPH oxidase releases ${\text{O}}_{2}^{-}$ to the phagosome lumen where through the action of super oxide dismutase (SOD) it is modified into H_2_O_2_, generating hydroxyl radicals, singlet oxygen, hypochlorous acid or chloroamines through myeloperoxidase activity (137). In the cytoplasm, increased expression of inducible nitric oxide synthase (NOS2 or iNOS) generates NO^−^ which can diffuse through the membrane to form nitrogen dioxide, peroxynitrite, dinitrogen trioxide, dinitrosyl ion complexes, nitrosothiols, and nitroxyl (138, 139). In the phagosome, ROS and RNS modify lipids, proteins and nucleic acids, culminating in bacterial death (65). In order to survive and replicate in the phagosome *M. tuberculosis* bacilli upregulate several antioxidant enzymes including superoxide dismutase C (SOD C), catalase-peroxidase-peroxynitritase T (KatG), and thiol peroxidase (TpX). SOD C detoxifies ${\text{O}}_{2}^{-}$ into molecular oxygen or hydrogen peroxide (65, 103), KatG neutralizes the NAPDH-derived peroxides pumped into the phagosome, and TpX generates resistance against macrophage generated RNS (140). As mentioned previously, TNF-α has a putative role during *M. tuberculosis* infection. *In vitro* studies with murine macrophages resembling early stages of infection, show that TNF-α-mediated iNOS and ROS induction significantly decreases *M. tuberculosis* growth (141). Similarly, mycobacterial-induced expression of miR146-a suppresses NO production through negative TNF-α regulation (31). Contrastingly, at later stages of infection, TNF-α induces necrosis of infected cells in the granuloma core leading to bacterial leakage and replication, making direct TNF-α cytokine therapy unsuitable for ROS and iNOS induction (51, 72, 76). Thus, ROS and iNOS inducers with no impact on cell death are a promising HDT approach for TB. Metformin is a FDA approved anti-diabetes drug shown to induce mitochondrial ROS production in *M. tuberculosis* infected macrophages and to decrease bacterial burden (35, 142). Furthermore, metformin has a positive anti-inflammatory impact decreasing *M. tuberculosis*-induced lung pathology (143) and positively regulates lipid metabolism (see below). In parallel, future therapeutic approaches focused on mir-146a repression in alveolar macrophages might also promote *M. tuberculosis* clearance or containment.

## Modulation of Lipid Metabolism and Macrophage Phenotype

*M. tuberculosis* efficiently modulates the macrophage glycolysis pathway and promotes ketogenesis and differentiation into permissive foamy cells (13). Foamy cells are lipid droplet rich macrophages, characteristic of chronic inflammatory diseases and infections (144). In macrophages, *M. tuberculosis* infection increases glucose uptake and redirects acetyl-CoA from the citric acid cycle to D-3-hydroxybutyrate synthesis, which signals through the anti-lipolytic G protein-coupled receptor GPR109A to induce lipid accumulation and lipid-body formation (13). Furthermore, *M. tuberculosis* cell wall lipids such as oxygenated ketomycolic and hydroxyl-mycolic acid activate TLR2 and the scavenger receptor MARCO to induce cholesterol uptake with sequestration and lipid droplet accumulation (145, 146) which can serve as a carbon source for *M. tuberculosis* to persist in nutrient limiting conditions (70, 144). These findings uncovered the cellular similarities of *M. tuberculosis* infection with other host metabolic diseases such as type II diabetes or hyperlipidemia and open the way to the use of anti-diabetic drugs and statins as possible HDT during TB (51, 73, 143, 147).

As previously described, metformin decreases *M. tuberculosis* replication in human macrophages through increased ROS production and bacterial killing (35). However, aside from its impact on macrophage oxidative state, metformin also reduces glycolysis efficiency, acetyl-CoA production and possibly ketogenesis in macrophages (36). A parallel therapeutic approach focuses on hypercholesterolemia drugs such as the statins that inhibit cholesterol synthesis and significantly decrease lipid accumulation (45). Despite the initial promising results in animal models treated with statins and antimycobacterial drugs (46, 47), a retrospective analysis with a national medical claim database failed to recognize any beneficial effect of this drug during *M. tuberculosis* infection (148). More retrospective studies and controlled clinical trials should help clarify the relevance of lipid accumulation and foamy cell differentiation in TB, and help determine if the currently available drugs for diabetes and hyperlipidemia can be effective HDT for *M. tuberculosis* infection.

## Modulation of Macrophage Iron Status

Iron is an essential element in all domains of life as an important cofactor for the synthesis and function of numerous proteins. Upon infection, *M. tuberculosis* must compete with the host for the same iron pool; *M. tuberculosis* strains mutated in iron sequestration genes show significantly attenuated growth *in vitro* and *in vivo* (149–151). In contrast, increased dietary iron or hemochromatosis is strongly associated with a worse disease prognosis during *M. tuberculosis* infection (152). Intracellular iron sequestration in macrophages is promoted by *M. tuberculosis* through two TLR-dependent redundant mechanisms targeting the host iron regulatory proteins hepcidin and ferroportin (23). Ferroportin is the only known iron exporter in mammals, highly expressed in macrophages, enterocytes and hepatocytes (153, 154). During iron overload or inflammation hepcidin secreted from macrophages and hepatocytes binds to ferroportin subsequently leading to its internalization and degradation. This process results in increased intracellular iron sequestration in macrophages, hepatocytes and enterocytes (155–157). Infection by *M. tuberculosis* in human macrophages directly downregulates ferroportin expression through TLR2 activation, and TLR4-induced endoplasmic reticulum-stress leads to hepcidin secretion which further decreases surface ferroportin. This decrease in ferroportin results in a significant increase in intracellular iron levels (23). Iron chelation therapy is a common strategy to avoid cardiac complications in hemochromatosis and thalassemia patients (158). During *M. tuberculosis* infection in human macrophages, iron chelation with the FDA approved deferiprone or deferasirox significantly decreases intracellular bacterial replication (159, 160). *In vivo*, deferasirox intraperitoneal injection during intravenous *M. avium* infection significantly decreases bacterial burden in the spleen but not in the lung or liver (161). Retrospective studies with hemochromatosis TB patients might unveil the interactions of iron chelation with standard anti-TB drugs regimen. Nonetheless, iron chelation therapy should be approached with care since it will exacerbate anemia due to chronic inflammation. A therapeutic alternative to decreasing iron availability to *M. tuberculosis*, and simultaneously decreasing the potential anemia, is direct hepcidin inhibition (162–164). Non-anticoagulant heparin significantly decreases hepcidin expression in hepatocytes (165–169), and heparin-mediated hepcidin inhibition decreases intracellular iron levels in human macrophages with pronounced effects in bacterial replication (49). Furthermore, blocking hepcidin function with specific antibodies is currently being tested for treatment of anemia with promising results (170), and could be expanded as a HDT for TB. Similarly, hepcidin blocking with a specific monoclonal antibody might decrease *M. tuberculosis* and other intracellular siderophilic bacteria replication in macrophages. Additionally, intracellular iron levels have recently been associated with macrophage polarization, with increased intracellular iron sequestration resulting in M2 phenotype (48). Consequently, hepcidin inhibition might not only decrease bacterial replication through nutritional immunity, but also modulate macrophage polarization toward M1 phenotype traditionally correlated with microbiocidal and proinflammatory activity. Further *in vitro* and *in vivo* studies will clarify the impact of hepcidin inhibition during *M. tuberculosis* infection, but the recent studies with other siderophilic bacteria strongly support the hepcidin-ferroportin axis as promising novel HDT for TB.

## Concluding Remarks

Tuberculosis remains a major public health concern in many areas of the world, and we are still far from achieving eradication. In today's globalized world, MDR- and XDR-TB are every nation's problem and need to be addressed. Novel HDT can help decrease MDR- and XDR-TB either by enhancing the effect of currently available anti-mycobacterial drugs, targeting new mechanisms, circumventing resistance, or by shortening treatment length which would facilitate patient compliance. Over the millennia that *M. tuberculosis* bacilli have infected humankind, host and pathogen have evolved mechanisms and relationships that greatly influence the outcome of infection. Understanding this evolutionary race and how host-pathogen interactions impact bacterial clearance or host pathology can lead the way to the rational development of new therapeutics that favor a host protective response. The host immune response to *M. tuberculosis* is a complex network of pro- and anti-inflammatory signals, and it is now clear that targeting a single aspect of the immune response with increased pro-inflammatory signals is not sufficient to treat TB. Most of the promising HDT presented here target many host-pathogen interactions and in some cases seem to induce both pro- and anti-inflammatory responses. As examples: heparin prevents *M. tuberculosis* invasion of alveolar pneumocytes and systemic dissemination, but also modulates macrophage intracellular iron levels, cytokine secretion and leukocyte recruitment. Hepcidin inhibition decreases intracellular iron levels, but also decreases lipid body formation and modulates cytokine secretion in macrophages. Similarly, metformin and vitamin D3 promote phagolysosome fusion and autophagy, while inducing anti-inflammatory cytokine secretion that prevents excessive lung pathology; and together, these compounds counteract multiple virulence mechanisms used by *M. tuberculosis* to evade the host immune response and establish infection. Likewise, the emergence of novel RNA delivery technologies will guide the development of RNA-based therapies targeting microRNA pathologically dysregulated during *M. tuberculosis* infection with broad metabolic targets.

Regardless of the preferred mechanism of action, HDT will most likely always be administered in combination with standard of care anti-mycobacterial drugs. Consequently, it will be important to assess possible drug-drug interactions between HDT and currently approved drug regimens. As example: rifampicin is well known to interact with corticosteroids and oral anticoagulants, that were here presented as possible HDTs.

HDT alone might never be enough to contain and clear *M. tuberculosis* bacilli in an active TB patient, but incorporating this treatment class will certainly increase the effect of our currently available anti-mycobacterial drugs, and might give our immune system the little push it needs to efficiently contain *M. tuberculosis* infection.

## Author Contributions

RA compiled the literature, conceptualized, wrote the manuscript, and prepared the figures. PG provided guidance, intellectual input, helped write the manuscript, and reviewed the manuscript. FQ provided guidance, intellectual input, helped write the manuscript, and reviewed the manuscript. All authors contributed to the article and approved the submitted version.

## Conflict of Interest

The authors declare that the research was conducted in the absence of any commercial or financial relationships that could be construed as a potential conflict of interest.
